# The interplay of food-related lifestyle and eating behavior in Italian women

**DOI:** 10.3389/fnut.2024.1338925

**Published:** 2024-02-06

**Authors:** Manal Hamam, Mario D’Amico, Daniela Spina, Giovanni La Via, Giuseppe Di Vita

**Affiliations:** Department of Agriculture, Food and Environment (Di3A), University of Catania, Catania, Italy

**Keywords:** food-related lifestyle, consumer behavior, women, cluster analysis, factor analysis

## Abstract

**Introduction:**

Women play a crucial role in food shopping and preparation, and their food choices have significant implications for their health and that of their families. This study aims to provide a perspective on women’s eating lifestyle, which has undergone significant changes.

**Methods:**

A factor analysis was conducted to assess the degree of involvement in food choices and the types of food items consumed among a sample of 399 Italian women.

**Results:**

Through cluster analysis, four segments were identified: hedonic food consumers, sustainable- and balanced-diet consumers, food experimenters, and no food fondness consumers. The results reveal a correlation between the degree of food involvement and the type of food consumed.

**Discussion:**

Furthermore, the food lifestyle of the sample is partially dependent on age. Individuals aged 25–28 years show more hedonic food consumption behavior, while the older age group (44–64 years) falls into the sustainable and balanced diet consumer cluster (the largest cluster) and the cluster of those who do not express definable food choices (no food fondness).

## 1 Introduction

Women represent a highly heterogeneous component of the population due to the strong diversity found regarding formative, work, migration, marital, and reproductive life trajectories (1). In addition, they play a significant role in society when it comes to food choices and food shopping; their influence is shaped by various factors, including cultural norms, gender roles, socioeconomic status, and individual preferences (2–5).

Over the decades, the role of women has changed radically as a result of a series of events that have seen them become more involved in the social and working world which have changed their lifestyles (6). Lifestyle is a social concept (7) shared by a group of individuals who hold comparable views on variables and are profoundly impacted by their concurrent requirements. It was mainly used primarily to assess activities, interests, and attitudes through which marketers have attempted to characterize homogeneous groups of customers and identify market trends (8, 9).

However, even when individuals share the same culture and social class, those affiliated with the same professions may not consistently adopt identical lifestyles. These variations can stem from demographic factors and individual expressions of identity, as well as integration factors such as age, education, income, and gender (10, 11).

The ambiguity of the lifestyle concept has raised concerns among experts over the years (12, 13) who have argued that lifestyles should be limited to certain aspects of life, such as food behavior (14), considered a complex system that is expressed through desires for self-actualization and representation and can certainly be considered an important component through which to describe the lifestyle.

The initial exploration of the elements constituting food lifestyles was undertaken by Brunsø et al. (15). They posited that a food lifestyle encompasses both declarative and procedural attributes. These attributes not only reflect an individual’s values but also indicate the qualities they may prioritize in food selection, providing insights into their food preferences.

Given the notable roles that women fulfill in modern society and the increasing attention they have received in recent studies, it is crucial to acknowledge that, while women may have a prominent influence on food choices, shopping, cooking, and household nourishment (16, 17), individual experiences and preferences can vary significantly (18). Furthermore, the lifestyle of women can exert a significant impact on their food choices and preferences. It is important to recognize that individual choices are subjective and can exhibit considerable variation; however, there are several common factors that contribute to this relationship (19–21). Food-related lifestyle can be viewed as “the system of cognitive categories, scripts and their associations that relate a set of products to a set of values” (22). It is a mental construct that describes the real behavior (23), thus demonstrating how product attributes are connected to the effects of food intake, transcending brands or goods, but that may be class-specific (24). In this respect, individuals vary not only in their level of participation with food (25), but also in the reasons behind their level of involvement with food, such as their desire to experiment with new foods and cooking techniques.

One of the most intriguing aspects is knowledge pertaining to gender variations in food relationships (26). Several studies have demonstrated significant gender differences in the relationship with food (27, 28). Since women are typically the primary food purchasers (29) and are often described as being especially attentive to sustainable and healthy diets (30–32) the main purpose of this study is to assess the eating lifestyle of a sample of Italian women through the correlation between involvement in food choices and the type of food consumed based on consumers’ stated preferences (33).

The theoretical framework employed in this study aligns with the model introduced by Brunsø et al. (34), which preserves the foundational principles of the established food-related lifestyle concept. Using their self-reported behaviors related to food engagement and dietary choices as key indicators, this model enabled the researchers to explore the food preferences and consumption patterns of a cohort of Italian women.

The results are intended to support food companies who, by identifying distinct consumer groups that adhere to different dietary patterns, can improve the effectiveness of their public campaigns and marketing strategies (21, 35).

## 2 Conceptual framework for food-related lifestyle: objective and research questions

A deductive model of food-related lifestyle was created in the late 1990s (22), which is considered more rigorous than the inductive approach prevalent in lifestyle research (36) and enables the description of eating behavior according to self-stated consumption preferences (37, 38).

Initially, the emphasis in food-related lifestyle was on tying innovation to the means–end perspective across several dimensions, such as shopping, meal preparation, and dining, which represents a wider approach compared to the innovation construct.

In several investigations the instrument has been updated, either by lowering the number of items or by tailoring it to the objective of the research, such as by making it more applicable to the kind of meal being evaluated (39, 40). It has been widely developed and successfully applied over the years to different European and non-European food cultures (41–43).

According to the theoretical model (44), food-related lifestyle manifests itself in several life domains, such as purchasing motives, quality aspects, cooking methods, shopping behaviors, and consumption situations, which are characterized by several latent dimensions that can be used to describe consumers’ lifestyles. The food-related lifestyle method views lifestyle as a cognitive mediator between life values, i.e., the fundamental end-states that individuals find desirable, and food-related perception and behavior (34). While it aligns more with Western cultures (45), it is widely accepted as a validated tool for international segmentation in the food sector (40). This method uses correlation between sets of psychographic attitudes and observable actions (46) and consists of three core dimensions: food involvement; food innovativeness; and food responsibility (34).

Given that the food-related lifestyle instrument has not previously been used to conduct a gender survey, this study is novel in terms of using a convenience sample of Italian women as the population of interest (47) to analyze the correlations between involvement in food consumption and the types of food they prefer.

Starting from the general hypothesis that eating habits and food lifestyles may coalesce in a survey specifically aiming to understand the behavioral features of female food consumers, the objective is to verify whether there is a relationship between the degree of food involvement and the types of foods consumed, which would enable us to describe the lifestyle of the sample surveyed according to the stated preferences of women consumers. Eating habits and food lifestyles are two related concepts; however, they have distinct meanings (48). Eating habits primarily focus on the specific behaviors and patterns of food consumption, encompassing the types of foods consumed, portion sizes, meal timing, frequency of eating, and preparation methods (49–51). Food lifestyles encompass a broader spectrum of attitudes, values, and practices related to food choices and behaviors (21, 52). Hence, it becomes intriguing to assess the food attitudes and motivations of females, with the aim of segmenting the broader target market into smaller, more homogenous groups or segments based on specific criteria such as environmental concerns, innovative food and cooking methods, healthy dietary preferences, or demographic characteristics.

Based on the above, this paper aims to answer the following research questions (RQs):

*RQ1*: Do environmental aspects still represent a fundamental motivation for women in their food choices today?

*RQ2*: Are women willing to experiment with innovative food and new culinary methods?

*RQ3*: Do women exclusively prefer healthy and nutritious foods?

*RQ4*: What are the most relevant foods in women’s diet today?

*RQ5*: Is it possible to identify homogeneous categories of female consumers to segment the food market?

*RQ6*: Are socio-demographic characteristics relevant in influencing food-related lifestyles?

## 3 Materials and methods

### 3.1 Data collection

An anonymous and structured questionnaire was administered, from March to April 2022, to a convenience sample of 399 Italian women via Google Forms (53–55). After a brief introduction, participants were asked to sign the informed consent form. The ethical review and approval for this study were waived due to the observational nature of the research, whereby consumer data were provided voluntarily. The questionnaire was circulated via major social networks, such as Facebook, WhatsApp, and LinkedIn, to gather data on participants’ eating habits and food lifestyles. While the sample size is deemed acceptable for multivariate analysis and model dependability (56), this sampling strategy necessitates that the findings be taken with care due to the reduced likelihood of generalizability. However, convenience sampling is a popular method in the scientific literature since the validity of the data is not compromised (57–60).

The questionnaire, aiming to collect a variety of qualitative and quantitative data on food consumption attitudes and purchase behavior, was divided into six parts: purchasing motives; quality aspects; cooking methods; ways of shopping; consumption situations; and socio-demographic characteristics. Respondents were presented with a series of coded options and asked to select the one that best reflected their opinion or behavior. The queries were structured as multiple-choice responses on a Likert scale with the intent of categorizing the respondents’ and their families’ attitudes and preferences.

To identify purchasing motives, the 15 items proposed by Brunsø et al. (34) were used, while maintaining the original concept of food-related lifestyle (19). This approach measures three basic dimensions of food-related lifestyle, namely food responsibility, food involvement, and food innovation; for each, the five items that would work best as indicators of the three constructed dimensions were identified. Responses were recorded using a seven-point Likert scale (1 = completely disagree; 7 = completely agree). Thereafter, self-reported eating behavior was analyzed using validated items assessing the frequency of: (a) consuming various sorts of goods; (b) utilizing extended time for cooking and baking; (c) using different types of shops; and (d) eating circumstances (34). Similarly, the survey comprised in part questions previously used in the validation of the food-related lifestyle instrument (19) and in part items created by Brunsø et al. (34) from scratch. In the second section of the questionnaire (quality aspects), respondents were asked how often they consumed items including fruits, vegetables, legumes, pasta, bread, meat, fish, sweets, and alcohol.

Responses were recorded using a seven-point Likert scale (1 = never; 7 = every day). In the third section of the questionnaire (cooking techniques), respondents were asked how often they spend more than one hour in the kitchen, such as during the week or on the weekend. In the fourth section of the questionnaire (shopping methods), respondents were asked how often they purchase food from supermarkets, retail outlets (e.g., greengrocer, fisherman, butcher, bakery), and the Internet. In the fifth section of the questionnaire (consumption situations), respondents were asked how often they eat meals in certain places, such as at home, at work, or at a restaurant.

The final section collected the social and demographic characteristics (Table 1) of the sample considering the following features: age group (18–24 years; 25–28 years; 29–43 years; and 44–64 years); education level; number of household members; and the presence of minors (<18 years old) in the household.

**Table 1:** Socio-demographic variables.

	Freq.	%
Age (years)		
18–24	78	19.55
25–28	102	25.56
29–43	106	26.57
44–64	113	28.32
Total	399	100.00
Education		
Primary and middle school certificate	18	4.51
High school diploma	171	42.86
University degree	175	43.86
Postgraduate degree (Master’s and/or PhD)	35	8.77
Total	399	100.00
No. of household members		
1	19	4.76
2	68	17.04
3	92	23.06
4	163	40.85
>4	57	14.29
Total	399	100.00
Presence of minors in the household (<18 years old)		
No	244	61.15
Yes	155	38.85
Total	399	100.00

### 3.2 Data analysis

The survey data were examined using inferential and multivariate statistical methods, including factor analysis and cluster analysis (61). The study first conducted two separate exploratory factor analyses (EFAs) on data blocks. Therefore, the dataset was divided into two subsets to separately summarize different information about the involvement of food consumption (EFA 1) and the type of foods (EFA 2) consumed by the sample examined. Subsequently, a separate EFA was performed on each block to reduce the dimensionality of the data and identify the main factors within each block. A complex or heterogeneous dataset with different blocks of variables may have different relationships or be influenced by different factors. By performing an EFA on each block separately, it was possible to capture the most relevant variations within each block. Following that, to verify the EFA1 model and determine the structure of the factors, confirmatory factor analysis (CFA) was performed. Finally, a cluster analysis was conducted on each block using the factor component scores as input variables to assign observations to clusters.

#### 3.2.1 Exploratory factor analysis

EFA was used to reduce the information contained in the original variables (62) into latent constructs, which were then utilized to identify homogenous consumer groups through cluster analysis.

EFA enables us to examine whether factors can describe the primary aspects of food involvement and consumption, summarizing the phenomenon’s description while minimizing information loss in terms of variance explained. This was accomplished by transforming the initial collection of the correlated variables into a new collection of orthogonal variables. Varimax rotation was used to facilitate the understanding of EFA findings and optimize the variance of the sum of square loadings (63). Therefore, factor loadings and explained variance in the results tables will relate to the rotated components.

This statistical technique was conducted separately for blocks of homogeneous variables (64), corresponding to two distinct sections of the questionnaire: the EFA1 to establish the underlying structure of the 15 items proposed by Brunsø et al. (34) pertaining to consumers’ food responsibility, food involvement and food innovation; and EFA 2 was conducted on 13 items pertaining to the type of food consumed by the sample. In the factor matrix analysis, we used 0.5 as the minimum value (65).

Kaiser–Meyer–Olkin (KMO) and Bartlett’s test, based on partial correlations between the variables, were utilized to validate the validity of the model (66).

The model’s fit was evaluated using the Kaiser–Meyer–Olkin (KMO) test, which is based on partial correlations between variables. The scores of the KMO test fall between 0 and 1. Low values indicate that the analysis is insufficient, since the correlation between pairs of variables cannot be explained by the variance shared by the whole collection of variables. Hence, it is advised that KMO test results not fall below 0.5, while findings over 0.7 are regarded as satisfactory (67).

Concerning the evaluation of the model’s validity, the Bartlett test is commonly utilized to test the hypothesis that the correlation matrix coincides with the identity matrix (68). When the Bartlett test is insignificant, the identity matrix may coincide with the correlation matrix; therefore, the factorial model may not be suitable.

#### 3.2.2 Confirmatory factor analysis

To validate the model and ascertain the structure of the factors, confirmatory factor analysis (CFA) was conducted. The CFA evaluates the fit of an *a priori* model containing the number of factors and the items that are assigned to them to the data.

The estimation method most frequently employed for CFA is maximum likelihood (ML).

The ML method operates under the assumption of a continuous scale, while the observed data adheres to an ordinal scale. However, Byrne (69) argues that the impact of considering ordinal data as continuous is negligible when there are more than five response categories, and the data are close to a normal distribution. The data utilized in this study satisfy the criteria, and the ML method was deemed a suitable estimation technique.

The evaluation of the fairness of fit was conducted utilizing the χ^2^ statistic. Nevertheless, due to the χ^2^ statistic’s susceptibility to large sample sizes (69) and in accordance with Hair et al. (70)‘s rule of thumb, an additional absolute and incremental fit indices were premeditatedly incorporated: Tucker Lewis Index (TFI), Comparative Fit Index (CFI), Root Mean Square Error of approximation (RMSEA), and Standardized Root Mean Square Residual (SRMR). Values exceeding 0.95 are regarded as indicative of a satisfactory fit for TLI and CFI, whereas values falling below 0.08 are indicative of a satisfactory fit for SRMR and RMSEA (71).

#### 3.2.3 Cluster analysis

To identify homogenous consumer groups, a cluster analysis using the k-means technique on factor scores derived from the EFA1 and CFA was conducted (72, 73). The k-means method is a nonhierarchical classification technique that permits the construction of clusters using an iterative procedure that minimizes Euclidean distances between centroids (74).

Using the pairwise distance matrix, the silhouette statistic was then calculated to determine the location of instances inside the clusters (75, 76). Comparing, for each example, the average distance from other cases in the cluster in which the case is placed to the average distance from the closest cluster yields silhouette width as a measure of cluster adequacy. A silhouette width less than 0 suggests a case that is a poor match with its cluster, while clusters are appropriately differentiated when this number is closer to 1.

Finally, to analyze how eating behavior varies within clusters by age group, a chi-square test was performed (77).

## 4 Results

### 4.1 Exploratory factor analysis (EFA) results

EFA 1 was carried out on 15 items related to consumer involvement in food consumption; the summary statistics of the variables used in the factor analysis are reported in Table 2. This yielded three different factors: environmentally friendly awareness; experimental orientation; and food indulgence.

**Table 2:** Summary statistics for consumer involvement in food consumption.

Item	Mean	Std. dev.	Min.	Max.
I just love good food	5.93	1.62	1	7
Eating and drinking are a continuous source of joy for me.	5.40	1.77	1	7
Decisions on what to eat and drink are very important for me.	5.32	1.70	1	7
Food and drink are an important part of my life.	5.03	1.85	1	7
Eating and food are an important part of my social life.	4.64	1.90	1	7
I like to try new foods that I have never tasted before.	4.84	1.95	1	7
I love to try recipes from different countries.	4.71	1.97	1	7
Recipes and articles on food from other culinary traditions encourage me to experiment in the kitchen.	4.32	2.00	1	7
I like to try out new recipes.	5.15	1.81	1	7
I look for ways to prepare unusual meals.	4.12	1.94	1	7
I try to choose food produced with minimal impact on the environment.	4.21	1.90	1	7
I am concerned about the conditions under which the food I buy is produced.	4.63	1.87	1	7
It is important to understand the environmental impact of our eating habits.	5.16	1.79	1	7
I try to choose food that is produced in a sustainable way.	4.51	1.89	1	7
I try to buy organically produced foods if possible.	4.24	1.88	1	7

^*^Items measured on a seven-point Likert scale (1 = completely disagree; 7 = completely agree).

The first factor draws information about consumer perceptions of environmental externalities due both to food production and consumption. This factor measures the weight that information related to, for example sustainable food production (0.9015) and environmental impacts (0.9007), have on food purchasing behavior. In view of the variables expressed, this factor was named “responsibility.”

The food attitudes of environmentally friendly consumers, also known as sustainable consumers, are characterized by a strong emphasis on the environmental impact of food choices. These individuals prioritize sustainability, ethical considerations, and the overall ecological footprint of the food they consume. The second factor tends to emphasize the aptitude to try new foods and recipes related to different culinary traditions. This factor explains a particular consumption pattern related to the willingness to try new foods. Consumers in this group show openness to new culinary experiences and trying new recipes in the kitchen (0.8369) from different countries (0.8592). In view of the variables expressed, this factor was named “innovation.” This factor includes consumers who are experimenting with new foods and can be described as adventurous eaters or culinary explorers. These individuals are open to trying unfamiliar or unconventional dishes, ingredients, or cuisines, actively seeking out novel culinary experiences. They may enjoy exploring different flavors, textures, and cultural influences, and often embrace the opportunity to expand their palate and discover new taste sensations. Experimenting with new foods can involve trying exotic dishes, experimenting with innovative cooking techniques, or exploring alternative dietary choices. This reflects their openness to stepping outside of their comfort zone and engaging in gastronomic exploration.

The third factor is characterized by information regarding the degree of involvement in food consumption. This factor emphasizes the importance of food as a source of joy (0.8225) and as an important aspect of life (0.8494). In view of the variables expressed, this factor was named “involvement.” This factor describes a consumer attitude characterized by a strong preference for pleasurable and indulgent food experiences.

EFA 2 was performed on 13 items related to the type of food consumed; the summary statistics of the variables used in the factor analysis are shown in Table 3. Consequently, the following four factors were identified: vegetable-based diet; carbohydrate-based diet; likes alcohol; and meat- and fish-based diet (Table 4).

**Table 3:** Summary statistics related to the type of food consumed.

Item	Mean	Std. dev.	Min.	Max.
Eat bread	5.74	1.51	1	7
Eat fish	4.51	1.20	1	7
Eat buttery/creamy sauces	3.17	1.56	1	7
Eat legumes	6.08	1.20	1	7
Eat vegetables	4.67	1.13	1	7
Eat salad	5.64	1.39	1	7
Eat fruit	5.96	1.42	1	7
Eat red meat	4.51	1.29	1	7
Eat pizza	4.45	0.88	1	7
Eat sweets, desserts, cakes	4.78	1.33	1	7
Drink wine	3.39	1.94	1	7
Drink beer	2.92	1.75	1	7
Drink milk	4.41	2.46	1	7

^*^Items measured on a seven-point Likert scale (1 = never; 7 = every day).

**Table 4:** Exploratory factor analysis (EFA 2) related to the type of food consumed.

	Vegetable-based diet	Carbohydrate-based diet	Likes alcohol	Meat and fish-based diet	KMO
Eat bread		0.5764			0.8030
Eat fish				0.6937	0.6320
Eat buttery/creamy sauces		0.5522			0.7003
Eat legumes	0.5022				0.6845
Eat vegetables	0.7940				0.7304
Eat salad	0.7332				0.6970
Eat fruit	0.7396				0.7643
Eat red meat				0.8267	0.5509
Eat pizza		0.7338			0.7354
Eat sweets, desserts, cakes		0.7585			0.6639
Drink wine			0.9061		0.5521
Drink beer			0.9139		0.5401
Drink milk					0.7854
Overall					0.6645
Bartlett test					0.000

^*^Blank cells represent abs(loading) < 0.5.

The first factor contains information about the consumption of fruits (0.7396), vegetables (0.7940), salad (0.7332), and legumes (0.5022), which explains a particular propensity to eat more vegetables. In view of this, the factor was named “vegetable-based diet.”

The second factor describes a particular consumption toward fancier foods, such as bread (0.5764), buttery and creamy sauce (0.5522), pizza (0.7338), and sweets, desserts, and cakes (0.7585), which explains a particular propensity to consume foods higher in carbohydrates (and fat). For this reason, this factor was named “carbohydrate-based diet.”

The third factor explains 13.2% of the variance and describes a particular propensity to consume alcoholic beverages, such as wine (0.9061) and beer (0.9139). Therefore, this factor was named “likes alcohol.”

Finally, the fourth factor describes higher consumption of red meat (0.8267) and fish (0.6937). Accordingly, this factor was named “meat and fish-based diet.”

Regarding the goodness of fit of the model, for both the factor analyses the values of the KMO test, 0.9149 in EFA 1 and 0.6645 in EFA 2 indicate that the variables are appropriate for factor analysis, which makes the model plausible for application. The high significance of Bertlett’s test result (0.000) in both EFAs also indicates that the variables contain a high amount of common information showing strong correlation between the variables, thus justifying factor analysis (Table 5).

**Table 5:** Exploratory factor analysis (EFA 1) on consumer involvement in food consumption.

	Responsibility	Innovation	Involvement	KMO
I just love good food			0.7530	0.9309
Eating and drinking are a continuous source of joy for me.			0.8225	0.9365
Decisions on what to eat and drink are very important for me.			0.7565	0.9473
Food and drink are an important part of my life.			0.8494	0.9073
Eating and food are an important part of my social life.			0.7411	0.9494
I like to try new foods that I have never tasted before.		0.8178		0.8852
I love to try recipes from different countries.		0.8592		0.8626
Recipes and articles on food from other culinary traditions encourage me to experiment in the kitchen.		0.8369		0.9184
I like to try out new recipes.		0.7045		0.9414
I look for ways to prepare unusual meals.		0.7772		0.9384
I try to choose food produced with minimal impact on the environment.	0.9007			0.8400
I am concerned about the conditions under which the food I buy is produced.	0.7740			0.9576
It is important to understand the environmental impact of our eating habits.	0.7694			0.9496
I try to choose food that is produced in a sustainable way.	0.9015			0.9468
I try to buy organically produced foods if possible.	0.8972			0.8413
Overall				0.9149
Bartlett test				0.000

^*^Blank cells represent abs(loading) < 0.5.

For this reason, the applied models and tests seem to be appropriate for analyzing the results (72, 73).

### 4.2 Confirmatory factor analysis (CFA) results

**Figure 1:**
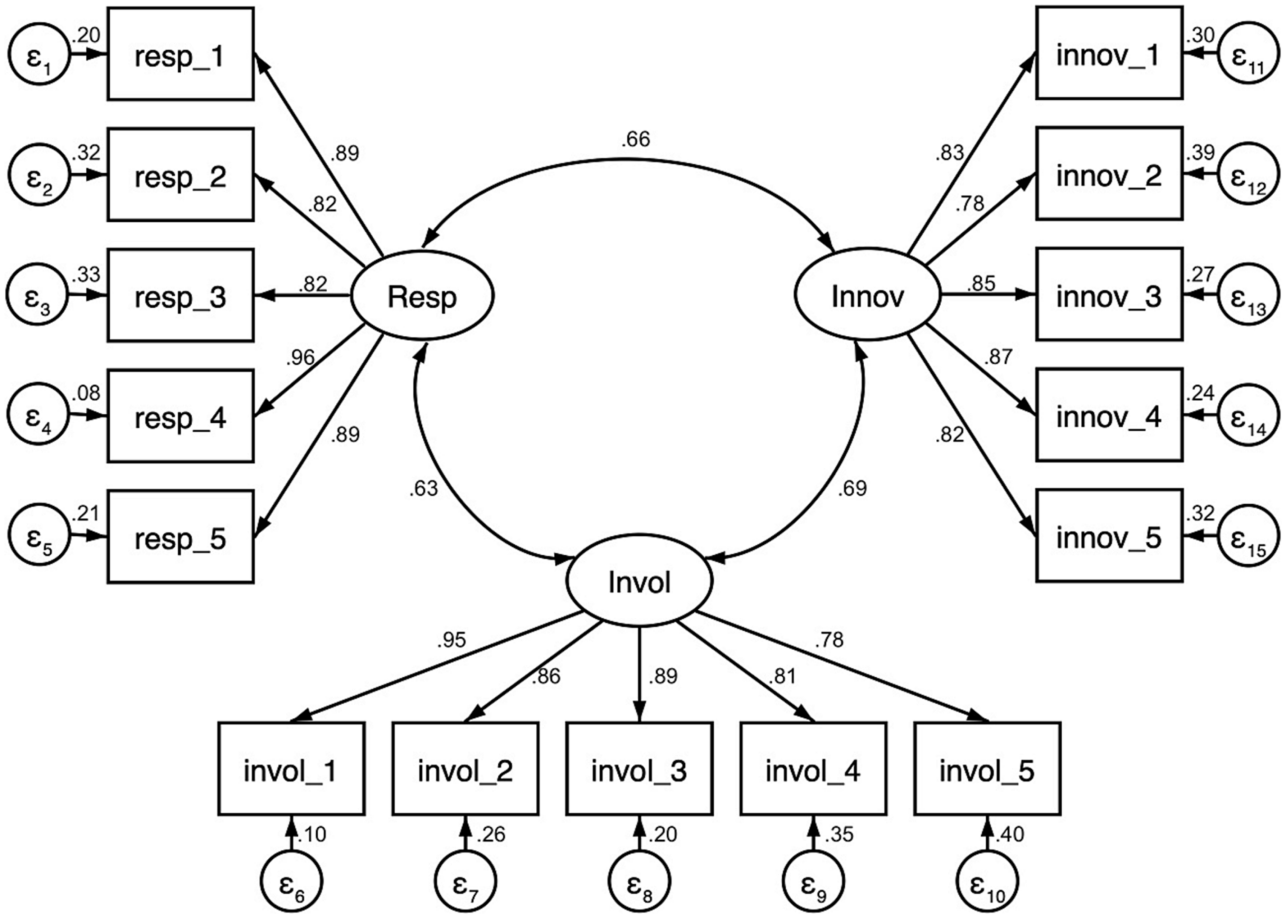
Measurement model for 15 items with standardized estimates.

The EFA1 was subjected to a confirmatory factor analysis (CFA) to reproduce the 15-item three-factor model. Based on the findings of EFA1 and as suggested by Brunsø et al. (34), it was postulated that items 11, 12, 13, 14, and 15 were associated with factor 1, while items 6, 7, 8, 9, 10, 11 were associated with factor 2, and items 1, 2, 3, 4, and 5 were associated with factor 3. According to the model’s results, each item and its corresponding factor produced statistically significant results. Indeed, the model appears to be exceptionally well-fitting overall, as indicated by the following indices: TLI = 0.987, CFI = 0.995, RMSEA = 0.047, and SRMR = 0.032. As an illustration, a strong positive correlation was observed between the factorial scores of EFA1 and CFA (responsibility = 0.90; involvement = 0.77; innovation = 0.80). In conclusion, the statistical analysis is corroborated by the constructs identified by Brunsø et al. (34). Consequently, the products serve as effective indicators for elucidating food responsibility, food involvement, and food innovation. Figure 1 illustrates the diagram representing the final measurement model incorporating the standardized estimates.

### 4.3 Cluster analysis results

This section presents the main results of the application of cluster analysis to the seven factors, the results of which led to the identification of four homogeneous clusters. The main characteristics of these clusters are shown in Table 6, in which the factorial scores in the centroids obtained by the k-means method are shown.

**Table 6:** Results of cluster analysis using the k-means method.

	Cluster 1 (Hedonic food consumers) (*n* = 69)	Cluster 2 (Sustainable- and balanced-diet consumers) (*n* = 99)	Cluster 3 (Food experimenters) (*n* = 150)	Cluster 4 (No food fondness) (*n* = 81)	Chi-square	*p*-value
Responsibility	−1.3732	0.5082	1.4177	−2.0773	8.16	0.043**
Innovation	−0.6249	0.2166	1.3723	−2.2738	22.14	0.000***
Involvement	0.3961	0.0450	1.1618	−2.5440	40.41	0.000***
Vegetable-based diet	−0.6639	0.1661	0.2756	−0.1480	18.86	0.000***
Carbohydrate-based diet	0.4102	0.1258	−0.1122	−0.2955	13.82	0.003**
Likes alcohol	0.0690	−0.5220	0.3771	−0.1190	4.02	0.259
Meat- and fish-based diet	0.2096	0.0951	−0.1725	0.0247	10.92	0.012**

*p ≤ 0.05; **p ≤ 0.01; ***p ≤ 0.001.

Four clusters emerged from the clustering results, carried out using the scores from CFA and EFA 2. All factors present themselves as significant in explaining the clusters, except for “likes alcohol,” which does not present itself as significant (Table 6).

The first cluster was labeled “hedonic food consumers” (*n* = 69), encompassing consumers who have a strong preference for hedonic foods, especially those rich in carbohydrates, such as bread and pizza. They have a particular affinity for sugary treats and tend to favor buttery and creamy sauces, as well as sweet desserts and cakes. This group includes individuals who derive pleasure and satisfaction from their food choices and eating experiences. They prioritize the sensory and emotional aspects of food, seeking pleasure, indulgence, and gratification through their food consumption.

The second cluster was identified as “sustainable- and balanced-diet consumers” (*n* = 99), referring to conscientious individuals who pay special attention to ecological aspects and follow a lifestyle related to a higher consumption of plant-based foods. This group includes consumers who prioritize both the health and environmental aspects of their food choices. They are mindful of how their food consumption affects their own well-being as well as the planet. These consumers strive to make choices that promote personal health and sustainability. Their focus is on maintaining a balanced diet that includes a variety of nutritious foods from different food groups, such as fruits, vegetables, whole grains, lean proteins, and healthy fats. They aim to meet their nutritional needs while ensuring a well-rounded intake of macronutrients and micronutrients.

The third cluster was defined as “food experimenters” (*n* = 150), representing consumers who have a penchant for culinary exploration and pay less attention to sustainable consumption practices. This group enjoys experimenting with new food recipes and cooking techniques and has a greater inclination toward trying novel and gourmet foods.

The fourth cluster was named “no food fondness” (*n* = 81), indicating consumers who have no preferences and are not particularly involved in their food choices. This class consists of individuals who do not have a strong preference or fondness for any specific types of food.

They may not derive great pleasure or satisfaction from eating and view food from a more practical standpoint, considering it primarily as a means to fulfill their nutritional needs rather than a source of enjoyment or excitement. They may not experience strong cravings or desires for specific foods and may be content with simple or basic meals.

In terms of socio-demographic characteristics (Table 7), only age seems to be significant, i.e., there seems to be variability in dietary lifestyle among the age groups. The first cluster (hedonic food consumers) consists largely of women who aged 25–28 years (37.68%). The second cluster (sustainable- and balanced-diet consumers) is largely composed of individuals aged 44–64 years (28.28%). In the third cluster (food experimenters), consumers aged 29–43 years seem to prevail (31.33%). Finally, in the fourth cluster (no food fondness), there is a slight prevalence of female consumers aged 44–64 years (38.27%). The choices of the lowest age group (18–24) do not seem to be as indicative in food choices compared to the other three age groups.

**Table 7:** Results of the chi-square test by age.

Age (years)	Cluster 1 (Hedonic food consumers) (*n* = 69)	Cluster 2 (Sustainable- and balanced-diet consumers) (*n* = 99)	Cluster 3 (Food experimenters) (*n* = 150)	Cluster 4 (No food fondness) (*n* = 81)	Total
18–24	15	20	27	16	78
	21.74%	20.20%	18.00%	19.75%	19.55%
25–28	26	25	36	15	102
	**37.68%**	25.25%	24.00%	18.52%	25.56%
29–43	21	26	47	19	106
	30.43%	26.26%	**31.33%**	23.46%	26.57%
44–64	7	28	40	31	113
	10.14%	**28.28%**	26.67%	**38.27%**	28.32%
Total	69	99	150	81	399
	100.00%	100.00%	100.00%	100.00%	100.00%

^*^Pearson chi-square = 18.1089 (*p-*value < 0.034).

## 5 Discussion

This section discusses the results concerning the eating habits and food lifestyles of women based on Brunsø et al. (34) model to understand the behavioral features of female food consumers. Considering the intricate nature of the factors that shape women’s identities and subsequently influence their choices, we have made an effort to provide a comprehensive analysis of all the results, starting from the factor analyses and concluding with the outcomes obtained from the clusters.

As regards the overall objective, we can confirm that there is a strong relationship between the degree of involvement in food choices and the type of foods consumed according to stated preferences, which enables us to describe the food lifestyle of the sample of Italian women. A person’s lifestyle, which encompasses various aspects of their daily routines, activities, and values, can significantly influence the types of foods they prefer; in this context, we found keyways in which lifestyle and women’s food preferences are related.

In response to RQ1, regarding the predisposition toward consciously consuming in a manner that respects environmental balance, the environmental awareness of our sample (with all its associated impacts) emerges, which appears to be highly consistent with the environmental sensitivity of the Italian female population (78, 79). Consequently, women who prioritize sustainability and environmental concerns in their lifestyle may have food preferences that reflect those values. This result appears to be strongly consistent with previous studies since women are more inclined to choose locally sourced, organic, or ethically produced foods, which may reduce the environmental impacts (80, 81).

Regarding RQ2, less widespread but no less relevant is the trend toward experimenting with new foods and recipes, both in the kitchen and at the table. The interest in innovative food options may be related to various aspects, such as convenience and sustainability, as well as to recipes or concepts specifically targeted toward women’s needs. This inclination, which has been widely found in the literature, reflects the high interest in innovative food by a certain proportion of women. Several authors have referred to the high interest in novel and innovative food products, such as irradiated or functional foods, foods with added functional ingredients, and foods with specific health benefits to support women’s well-being (82–84).

In response to RQ3, regarding whether women exclusively prefer healthy foods, a noticeable inclination among women toward consuming carbohydrate-rich and sweetened foods can be observed. Specifically, although hedonism is a more pronounced characteristic among men, while women usually make healthier dietary food choices (2, 85, 86), our study evidences that women, albeit to a limited extent, also appear to be attracted to the pleasure of good cuisine and dining, thus also preferring unhealthy food. This appears to be quite consistent with some previous studies, wherein a certain proportion of women tend to make unhealthier food choices; in particular, women with lower educational attainment tend to have an unbalanced diets and have lower food involvement (87, 88).

In response to RQ4, regarding the types of food consumed, four distinct eating patterns emerged: vegetable-based diet; carbohydrate-based diet; likes alcohol; and meat- and fish-based diet. The first pattern (vegetable-based diet) encompasses individuals who follow a vegetarian lifestyle. This finding aligns with existing literature, as it has been widely reported that some women opt for a vegetarian or vegan lifestyle, avoiding animal products in their diets. Additionally, prior studies have often associated females with a higher intake of vegetables and fruits (80). These individuals may prioritize plant-based foods, such as fruits, vegetables, legumes, whole grains, and plant-based protein sources, being prosocially motivated to follow such a diet (4).

The second factor shows the importance of the carbohydrate-based diet factor. This result is in line with previous research that considers high-carbohydrate foods, such as bread and pasta, tasty and able to provide useful nutrients for human nutrition (50, 89). However, this factor also includes unhealthy foods, such as desserts and cakes. The consumption of such products, that consumers believe to be unhealthy (87), is consolidated and the overconsumption of highly sugary foods still represents a widespread model even among women (90–93).

Equally important is the animal-derived protein consumption pattern (meat- and fish-based diet). Despite the recent growing interest in plant-based protein, the consumption of animal-derived protein remains prevalent in many parts of the world: animal-derived protein sources, such as meat, poultry, fish, eggs, and dairy products, are still traditional sources of protein in human diets (94–96).

Finally, women’s inclination to consume alcoholic beverages did not show statistical significance. However, even though women generally consume fewer alcoholic beverages than men (97), a bias toward beer and wine was found among female respondents. This result is in line with previous studies on female consumers’ preferences for beer and wine (98–100).

Regarding RQ5, when considering food preferences, it is challenging to categorize women into specific clusters as individuals have diverse tastes and preferences. However, the survey aimed to explore potential homogeneous market segments, revealing certain broad patterns and clusters of food preferences among women already found in previous FRL applications (20, 34, 40, 101). In this regard, it was observed that four main consumer groups emerged: hedonic food consumers; sustainable- and balanced-diet consumers; food experimenters; and no food fondness consumers. This implies that generalizable categories can be found even for the complex feminine universe.

First, a strong tendency to be high-intensity sustainable consumers emerged; women are often more conscious of the environmental impact of their consumption habits and strive to make choices that align with their values. This is strongly consistent with what prior literature has evidenced, namely that women may prioritize sustainable food products (79, 102, 103).

The emergence of the identified cluster validates findings from prior Food-Related Lifestyle (FRL) applications, which also identified segments of rational consumers (34, 40). These segments are characterized by a heightened interest in health and product information, a preference for shopping at specialized shops and markets, a tendency to consume organic food, meticulous scrutiny of product labels, and a prioritization of taste and healthiness over convenience and brand (104).

As regards the hedonistic groups, our study evidenced how some women may prefer comfort foods or indulge in specific treats or high-carbohydrate foods on occasion. These preferences may include sugar-sweetened desserts or sweets, or pizza or grain-based foods (that are high in calories), which certainly provide emotional satisfaction. In this context, food indulgence can be induced by specific emotional states, such as nostalgia or anxiety (3, 105). This group of consumers seems to be characterized by being highly indulgent in relation to unhealthy food.

Previous FRL applications (20, 40, 101, 106) identified consumer segments inclining toward hedonism, or the pursuit of delight and enjoyment, which are corroborated by the formation of this cluster (20).

As regards experimental behaviors in foods, we included all those respondents willing to try new and unfamiliar foods, ingredients, or cooking techniques. This group is characterized by curiosity and openness toward exploring different culinary experiences and expanding one’s food preferences. People with experimental behaviors in food enjoy seeking out novel and unique food experiences, and they may actively seek opportunities to try new dishes, cuisines, or food combinations. This behavior is often associated with a sense of enjoyment in discovering new tastes and textures. Experimental behaviors in food can contribute to a diverse and varied food repertoire, as individuals continuously explore and expand their culinary horizons. Although these attitudes, differentiated by gender, are not so common in the literature, our study is consistent with the findings of prior studies providing specific insights into women’s tendency to experiment with novel food (84, 107, 108).

Previous FRL applications have identified adventurous consumer segments (34, 40, 106) that exhibit the following characteristics: a penchant for social and self-validating food, an inclination toward quality, pleasure derived from preparing meals and products that are novel, and a demand for innovation (34, 40, 106) in terms of products and meals.

Finally, as regards the last group (individuals with no fondness for food), these individuals can be described as consumers having a relatively neutral or indifferent attitude toward food (52). They may not derive much pleasure or interest from different tastes or culinary experiences. For them, food is primarily seen as fulfilling their basic nutritional needs rather than being a source of enjoyment. They seem not invest much time or effort in culinary experiences or experimenting with different types of cuisine. This low food involvement has been previously detected in the literature concerning women who focus on practicality and efficiency rather than indulgence or variety, as well as among those not prioritizing eating a well-balanced diet, which is common among women with a low educational level (80, 109).

The cluster in question has been characterized in prior FRL applications through the identification of segments of uninvolved consumers (34, 40, 52, 106, 110) and careless consumers (40, 104): the former refers to individuals who are less inclined to shop in specialized shops or at the market, disregard organic food labels, cook infrequently, and do not adhere to a strict food regimen. The latter cohort consists primarily of snack and convenience food consumers.

Finally, in response to RQ6, regarding the socio-demographic characteristics of the sample, although education and the number of household members were not found to be relevant in influencing food-related lifestyles, it is noteworthy that food preferences have different directions by age group. Our results confirm previous findings in numerous studies showing that older individuals are more likely to make healthier and more sustainable food choices, while younger consumers are inclined to experiment more and are less inclined to make more conscious environmental food choices (111–115).

## 6 Conclusion

### 6.1 Main results and implications

Women are often considered the primary food shoppers in many households. They are frequently responsible for planning meals, creating shopping lists, and purchasing groceries for the family. This role places them at the forefront of decision-making regarding food choices. Within this context, and given the significant influence that women have on food choices and preferences in modern society, the present work aimed to explore the eating lifestyle of Italian women by assessing the degree of association between involvement and eating habits.

Summarizing the main findings of this study, conducted through factor analyses and a subsequent cluster analysis, a direct relationship emerges between the degree of involvement and women’s food preferences. Drawing inspiration from factor analysis, the extreme importance of environmental awareness (conscious choice), a strong inclination toward food experimentation, and a certain relevance of hedonism leading to food choices that are not always necessarily healthy can be observed.

Regarding the cluster analysis results, which generated four different groups, the three patterns obtained from the factor analysis are confirmed, with the addition of a completely disinterested category that seems to have little inclination toward choices determined by taste, preferences, or food-related lifestyles.

The results of this study thus add incremental knowledge related to marketing to women, as they provide further insights into the role women play in directing the diets of family members and shaping society’s food preferences based on food lifestyle theory.

In addition, there are also social implications related to female food consumers. Specifically, our study shows that, as well as many women being involved in consuming sustainable food and interested in balanced diet, there are any female consumer segments attracted by unhealthy foods or even totally disinterested in what they eat.

In this regard, these results may have implications for public health nutritional initiatives that could be formulated to enhance the well-being of women.

To design strategies to encourage young women to adopt healthy eating habits, social marketing can, in fact, assist policymakers in comprehending the target audience and customizing messages for distinct segments.

In accordance with their needs, beliefs, and intentions, segmentation thus determines which groups are most susceptible to persuasion regarding the adoption of the desired behavior.

### 6.2 Limitations and future research

One potential limitation pertains to the utilization of a convenience sample, which calls for caution when generalizing the findings to the broader population. However, considering this study as an initial exploratory analysis, the chosen sample can be considered suitable for examining the food-related lifestyle of Italian women.

Another aspect is that the present study may appear inconsistent with modern gender theories, which challenge strict categorizations and recognize blurred distinctions between genders, acknowledging intermediate stages. These theories call for a nuanced understanding of gender differences, including in food preferences shaped by diverse social and cultural influences. However, we argue that it is still meaningful to discuss gender differences in food preferences and choices, even in light of these new theories. This is because food preferences and choices result from a complex interplay of biological, social, cultural, and experiential factors, including those associated with gender.

Additional studies could be carried out by including different countries of EU to detect differences and contact points among European women.

In addition, it could be interesting to add psychometric constructs to identify more, and more complex, clusters. Specifically, these clusters are not exhaustive, and individual preferences can vary widely.

Finally, it is important to note that food preferences could change over time due to factors such as personal experiences, health concerns, environmental influences, and evolving dietary knowledge.

## Data availability statement

The raw data supporting the conclusions of this article will be made available by the authors, without undue reservation.

## Ethics statement

Ethical approval was not required for the studies involving humans because for this type of study, our university does not require approval from the ethics committee but only the informed consent of the participants is necessary. The studies were conducted in accordance with the local legislation and institutional requirements. The participants provided their written informed consent to participate in this study.

## Author contributions

MH: Conceptualization, Data curation, Formal analysis, Investigation, Methodology, Software, Writing – original draft, Writing – review & editing. MD’A: Conceptualization, Funding acquisition, Supervision, Validation, Writing – review & editing. DS: Conceptualization, Formal analysis, Investigation, Writing – original draft, Writing – review & editing. GLV: Supervision, Validation, Writing – review & editing. GDV: Conceptualization, Investigation, Methodology, Supervision, Validation, Writing – original draft, Writing – review & editing.
